# Enhanced Parkinson's disease prediction using LDEFS feature selection and Mamdani fuzzy neural network

**DOI:** 10.3389/fnagi.2025.1665590

**Published:** 2025-12-09

**Authors:** M. Vijayalakshmi, B. Dhiyanesh, D. Viji, P. Saranya

**Affiliations:** 1 CSE Computing Technologies, SRM Institute of Science and Technology, Kattankulathur Campus, Chennai, Tamil Nadu, India; 2 CSE ETech, SRM Institute of Science and Technology, Vadapalani Campus, Chennai, Tamil Nadu, India; 3 CSE Computational Intelligence, SRM Institute of Science and Technology, Kattankulathur Campus, Chennai, Tamil Nadu, India

**Keywords:** Parkinson's disease, neural network, impact scaling rate, *Z*-score normalization, neurodegenerative, early disease detection, medical diagnosis

## Abstract

**Introduction:**

Parkinson's Disease (PD) is a progressive neurodegenerative disorder caused by the degeneration of dopaminergic neurons, leading to impairments in speech, motor control, and cognitive functioning. Although recent computational models have improved diagnostic accuracy, many still depend on manual intervention, fail to account for exercise-related patterns, and may contribute to disease misclassification. There is a growing need for an automated and highly reliable predictive model capable of handling large volumes of clinical data.

**Methods:**

A Parkinson's disease dataset was obtained from an online public repository. To improve data quality, Z-Score Normalization (ZSN) was applied to minimize noise and eliminate irrelevant records. The Disease Affect Scaling Rate (DASR) technique was then employed to quantify and rank the influence of disease-related features. Feature selection was performed using the proposed Logistic Decision Exhaustive Feature Selection (LDEFS) approach to extract the most significant disease indicators. Finally, the Mamdani Fuzzy Neural Network (MFNN) model was developed for PD prediction using the optimal feature subset.

**Results:**

The proposed LDEFS–MFNN framework demonstrated superior detection capability compared to existing approaches. Experimental evaluation showed a prediction accuracy of 95.8% and an F-measure of 95.3% for early PD detection, outperforming previous machine-learning classifiers reported in the literature.

**Discussion:**

Results confirm that the integration of exhaustive feature ranking with fuzzy neural modeling enhances PD prediction performance while minimizing the need for human intervention. The inclusion of exercise-related patterns and optimized feature weighting leads to improved robustness in classification. Therefore, the proposed system offers a reliable and scalable solution for early Parkinson's disease diagnosis and has strong potential for clinical deployment.

## 1 Introduction

In addition to being a highly prevalent and impactful disorder, Parkinson's disease (PD) affects a significant portion of the population. Tragically, many lives are lost due to a failure to identify and diagnose the disease promptly. Early detection of cardiovascular disease risk can significantly reduce the risk. As data from the healthcare industry grows, machine learning (ML) techniques can predict disease according to patients' side effects. The current level incorporates ML algorithms for specific prediction objectives determined through a comprehensive feature evaluation process (Giannakopoulou et al., 2022). An influential organ of the human body is the brain, which causes disorders. Predicting failure incidence through monitoring data is difficult in the medical field. Machine learning (ML), the Internet of Things (IoT), and deep learning (DL) are effective solutions for healthcare challenges (Nasser and Mahmood, 2021), biological communities, and clinical management. Moreover, practical interpretation of clinical data helps in disease diagnosis.

A higher incidence of PD is observed among the elderly, making it the second most prevalent neurodegenerative disease (Jayaram and Senthil Kumar, 2021). The disease takes a long time to detect and is often incurable, resulting in high mortality rates worldwide. It is a chronic, evolving neurological disorder that impairs movement (Shahid, 2020). Anitha et al. (2020) PD disease affects the patient's quality of life, makes it challenging to get along, and worsens the economic crisis due to high medical expenses. PD leads to various motor and non-motor symptoms that increase in severity as the disease progresses. Sajal et al. (2020) it is common for PD patients to experience slowed movements, difficulty with shaking, and trouble walking in the early stages. Various symptoms are associated with late-stage PD, including depression, anxiety, and dementia.

Medical data has increased recently, requiring efficient classification and prediction algorithms. Therefore, ML technologies are essential in clinical diagnostics and are widely employed in bioinformatics (Saeed et al., 2022). Furthermore, Feature Selection (FS) is crucial in interpreting clinical data. As a result of this technique, fewer features are included in the initial set of features that are being evaluated. It removes irrelevant or redundant features without losing information, thereby reducing training time.

However, previous studies used ML techniques like XGBoost and Support Vector Machine (SVM) for PD detection (Mohesh et al., 2022). Nonetheless, these methods produce less prediction accuracy and take more time and complexity during classification. An earlier and more accurate diagnosis is of significant help for effective treatment and disability limitations for PD identification (Chakraborty et al., 2016). Kaur et al. (2017) Research conducted in this study contributes to diagnosing and analyzing diseases at an early stage through DL. As a first step, null values are removed through preprocessing. Using the *Z*-score normalization method, these data are used to determine the features associated with the disease. This study uses the Logistic Decision Exhaustive Feature Selection (LDEFS) technique and MFNN (Mamdani Fuzzy Neural Network) for PD detection to eliminate the low-accuracy PD detection problem.

### 1.1 Objective of this study

- The proposed *Z*-Score Normalization (ZSN) technique minimizes noise and irrelevant records. After that, we use the Disease Affect Scaling Rate (DASR) technique to identify the disease effect level.
- Based on the disease severity level, we choose the most significant features of the disease using the LDEFS technique. Later, our proposed MFNN algorithm with a softmax activation function predicts PD based on selected features.
- To increase the precision, classification accuracy, recall rate, and the F-measure with redundant time complexity. A high performance indicates that the proposed disease identification system will improve the performance of existing systems in terms of validation.

Parkinson's disease is assessed using various diagnostic methods, such as classification, accuracy, specificity, and sensitivity analysis. The newly proposed predictive model aims to enable early diagnosis and detection of PD to determine whether someone is affected by the disease.

## 2 Related works

An ANFIS algorithm based on IoT and cloud environments was presented as a novel method for detecting PD using ML (Khan and Algarni, 2020). Adaptive Neuro-Fuzzy Inference System (ANFIS) learning is robust to gradient-trapped local minima. Using Modified Salp Swarm Optimization (MSSO) to optimize learning parameters can improve ANFIS results. Furthermore, another study examined the ANFIS method and a hybrid algorithm for PD identification. At the edge of local community gateways, fog computing gathers, analyzes, and transmits data in real time (El-Hasnony et al., 2020). Despite this, most methods produce lower prediction performance. The author focused on the previous problem; therefore, the study introduced fuzzy neural systems for PD identification. A fuzzy neural system (FNS) is proposed to provide structural and learning mechanisms. The innovative approach aims to enhance the system's effectiveness and effectively distinguish between healthy individuals (Rahib and Abiyev, 2016).

In addition to the ANFIS-modified Glowworm Swarm Optimization Algorithm (M-GSO) for the diagnosis of cancer-causing symptoms of the predictive model for diagnosing neurodegenerative disorders such as glaucoma, Parkinson's, and neural tube syndrome (Balasubramanian and Ananthamoorthy, 2021), ANFIS-M-GSO can be used for detecting cancer-causing symptoms. However, predicting accurate results from the collected dataset is still a challenge. The novel focuses on past issues; therefore, the study introduces the Fuzzy Inference System (FIS) for PD classification. The proposed system can monitor voice signals using a telephone system. This method can provide remote confirmation of a clinical PD diagnosis and be used with supplementary diagnostic tests (Haq et al., 2019). However, this work requires accurate disease prediction. To address the previous problem, the study employed feature selection based on SVM for PD prediction. L1-normative SVM feature selection can be applied to select relevant and highly relevant features of PD and healthy individuals to achieve accurate target classification. PD dataset features can be generated using the L1-norm SVM based on the feature weights (Faouzi et al., 2022).

Furthermore, the article introduced an ML-based technique for PD detection with genetic data. Clinical risk factors using genetic variants and regressors with prior Impulse Control Disorders (ICDs) and recurrent neural networks trained on three logistic relationships to identify ICDs (Zhang et al., 2020). The study focused on PD detection using ML techniques from impaired gait patterns. It improves accuracy and minimizes latency when predicting PD. A combination of impaired gait and typical Freezing of Gait (FoG) detection features was determined based on the signal's steps. The study used FoG and AdaBoost to develop prediction models. Examine whether the FoG can be better predicted depending on the impaired gait features (Sabo et al., 2022).

Similarly, the study used video-based gait features in adults with PD. The data was examined, encompassing the mean gait variables for each participant's steps. Various outcomes were derived, including spatial and contrast gait measures, the minimal disparities between two camera heights, and gait variables obtained using video-tracking algorithms, specifically AlphaPose and Detectron (Yoon and Li, 2019). Another study presented Transfer Learning (TL) for telemonitoring PD. This limitation can be overcome by the Positive TL (PTL) method. PTL drives the development of robust novel mechanisms and can be applied to provide an in-depth theoretical analysis of adverse transfer risks and conditions. However, this method faces many challenges in predicting PD. The novel focused on predicting PD and Multiple Sclerosis (MS) based on vision features using deep learning approaches. Individuals with MS, PD, and Healthy Older Adults (HOA) used a data-driven approach to characterize generalizable improvements in imagery with different walking tasks. A detailed quantitative comparison of ML and DL algorithms with traditional 16-contrast methods is provided (Kaur et al., 2023).

Another study applied the DL model to PD identification through emotional facial expression. First, PD can approximate patients' appearances and can be used to create virtual human images. Next, the quality of the images depicting facial expressions must be synthesized and evaluated. Thirdly, we train the classifiers on a deep feature extraction tool developed on a combination of the entire dataset (Huang et al., 2023). The study proposed ML-based techniques such as Naive Bayes (NB), Support Vector Machine (SVM), K-Nearest Neighbors (KNN), Artificial Neural Networks (ANNs), and Decision Tree (DT) to improve disease prediction. Classification accuracy increases, and processing time is reduced through feature selection algorithms. They are also helpful for learning effective practices in model estimation and hyperparameter tuning (Li et al., 2020).

Similarly, another study conducted by the same researchers used ML techniques to predict chronic kidney disease. A complete set of features serves as the basis for feature selection for each classifier. Wrapper methods are calculated by combining features, least absolute summation, selection operator regression, synthetic minority, and others (Chittora et al., 2021). Disease prediction is challenging. The study used ML based on means, DT, and random forest to combat previous challenges. Based on 183 healthy individuals and 401 early-onset PD patients, the proposed DL model was compared with a predefined 12-ML dataset and ensemble learning methods. This model exhibits high detection performance and high accuracy (Camps et al., 2018).

DL methods can be used to diagnose FOG episodes in PD patients, based on the best available literature. A representation strategy is carried out using the updated spectral data and information in the signal from previous and current windows in this model. However, patients' quality of life declines compared with FOG episodes, which are less severe (Naghavi and Wade, 2019). The authors proposed that freezing of gait can be predicted to define the most practical combination of features. Data collected from lower-limb accelerometers uses statistical analysis of window elements and discriminates in standard practice (Del Din et al., 2019). The author proposed examining common associations between non-clamped fallers and non-fallers in independent-living practice. Macro variables with ambulatory bouts are diagnosed with fewer and less variable pathologies than those without falls (Romijnders et al., 2021). Participants were tracked using a motion-capture system during three distinct walking tasks. The inertial measurement units (IMUs) were used to attach markers to the heel and toe of the shoe to track movement accurately. A pair of IMUs was placed on each shank on the lateral sides. It has been proposed that computer vision-based models be developed to enable non-invasive video recordings to be observed, 3D body skeletons to be extracted over time, and MDS-UPDRS scores to be predicted based on the movement of the subjects. Neurodegenerative disorders lead to PD. This condition results in postural instability, resting tremors, rigidity, and bradykinesia (Lu et al., 2020). The author proposes that a pipeline of 40-foot-worn Inertial Measurement Unit (IMU) data from recently published PD patients can be analyzed. Aside from level walking, stair ascents, and descents, it extracts objective gait parameters. However, the prevalence is exceptionally high in patients with neurological diseases such as PD (Roth et al., 2022). Based on IMU data, the existing level is discovered in the pipeline to sequence experiments in unsupervised real-world procedures. However, unsupervised gait tests can be analyzed, and patients' reliable test notes can be relied upon (Ullrich et al., 2021). Due to non-related feature selection during the disease identification process, the results are produced with low precision and a low recall rate. Another study (Hassan and Ahmed, 2023) is concentrated on predicting PD progression using a non-invasive method. Feature engineering and data augmentation methods address the data imbalance issues. Additionally, an ensemble-based stacked model showcases the efficiency for PD using voice input. In this study (Shen et al., 2025), propose an interpretable artificial intelligence framework that can analyze speech features and achieve high diagnostic accuracy, while ensuring the interpretation of results for clinical use. Similarly, another article (Simone et al., 2025) focused on interpretable models for speech-based PD diagnosis and demonstrated that transparency of model results can improve clinical reliability.

Hassan et al. (2025) proposed a fusion technique integrating multiple algorithms with explainable AI to accurately predict Gestational Diabetes Mellitus (GDM). The hybrid model improved diagnostic precision and interpretability, offering better clinical insights for maternal healthcare. Hassan and Ahmed (2023) developed a non-invasive method leveraging voice input analysis to predict PD progression. The approach effectively captured vocal biomarkers, enabling early detection and continuous monitoring of disease severity.

Hassan et al. (2022) introduced an explainable ML framework for predicting preterm birth risk. Their model emphasized feature interpretability to help clinicians understand risk factors influencing premature delivery. Gulzar Ahmad et al. (2025) designed an IoT-based smart wearable belt for tracking fetal kicks and movements in expectant mothers. The system employed embedded sensors and real-time analytics to ensure continuous fetal monitoring and early anomaly detection.

### 2.1 Problem consideration factor identification

Given the vast amount of clinical data available in the healthcare sector, ML algorithms become imperative to making accurate predictions regarding PD. In the context of feature selection, it is crucial to identify and prioritize the most significant features carefully. Due to a lack of feature analysis, high-dimensional risk profiles failed to predict the likelihood of events occurring. A hybrid model introduces more features, leading to lower feature selection accuracy because irrelevant features degrade classification accuracy in the model. So, the increasing misinformation in features and non-relation weights is not adequately used to determine the result properly for early diagnosis.

## 3 Proposed methodologies

**Figure 1:**
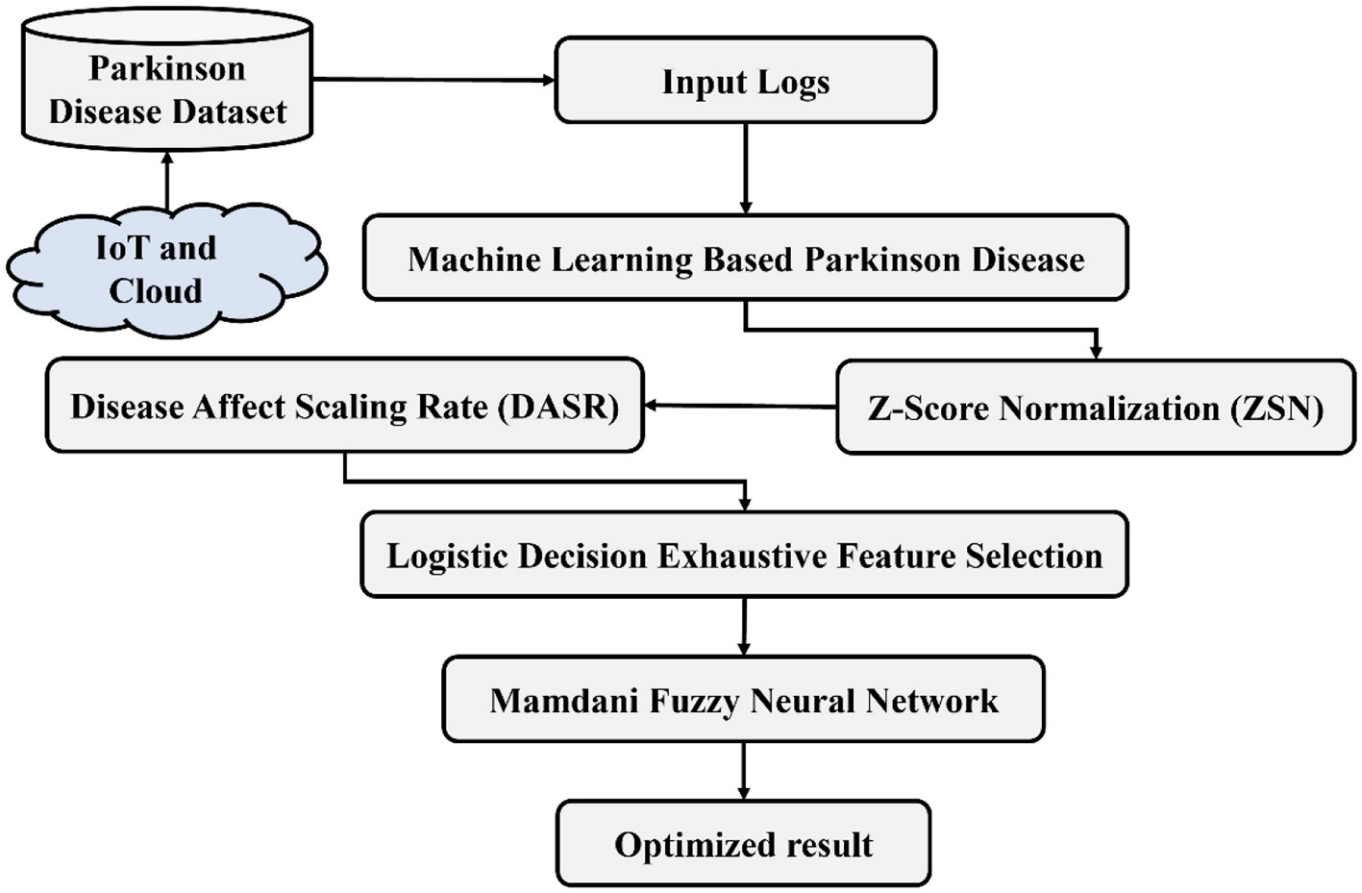
Proposed architecture of MFNN algorithm.

This section elaborates on cloud-based PD prediction using the LDEFS technique and MFNN approaches. The proposed method involves four stages: (i) preprocessing, (ii) attribute weight analysis, (iii) feature selection, and (iv) classification. Figure 1 presents an architectural diagram illustrating the utilization of ML techniques for PD prediction. Initially, the proposed *Z*-score normalization technique was used to reduce missing values. Then, we apply the DASR method to analyze the disease-feature weights. Afterward, the LDEFS method selects the best features based on weight. Thus, the MFNN algorithm successfully categorizes PD.

### 3.1 Data collection

The PD dataset was obtained from the Kaggle repository. It includes features such as average, maximum, and minimum vocal fundamental frequency, pitch perturbation quotient, noise-to-harmonics ratio, harmonics-to-noise ratio, and status. Dopamine deficiency is one of the hallmarks of PD, which is a neurodegenerative disease that affects the brain. This manifested as reduced motor skills, such as tremors and rigidity.

Speech is often markedly affected, including dysarthria (difficulty speaking), hypophonia (reduced volume), and monotonicity (decreased range of vocal tones). Furthermore, cognitive impairment and mood changes can occur, increasing dementia risk.

**Figure 2:**
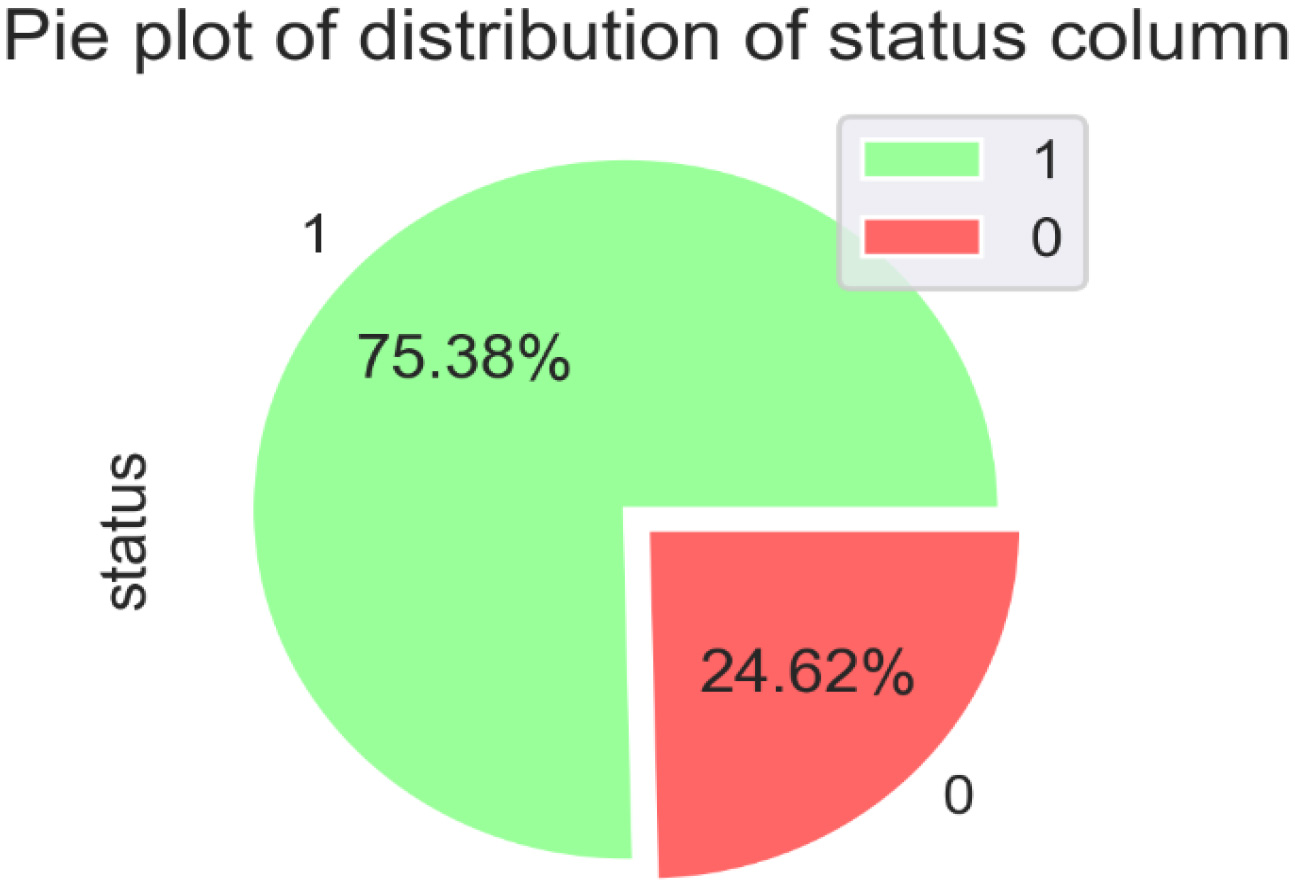
Distribution of the dataset status.

Figure 2 compares individuals diagnosed with PD with those considered healthy. The diagram uses a binary representation: 0 signifies individuals without any health conditions, while 1 represents those affected by PD.

### 3.2 *Z*-score normalization

Parkinson's disease cumulative scores may not be accurate because they contain missing values. Hence, the *Z*-score normalization method is applied to correct the dataset. The normalization algorithm removes irrelevant and missing values based on the mean and standard deviation.

The following equation estimates the mean values *M*_*n*_,


$$\begin{array}{l} Mean\;\left(M_{n}\right)=\frac{\sum_{c}^{r}P_{R}}{N_{R}} \end{array}$$ (1)

Where *P*_*R*_ denotes present records in the dataset; *N*_*R*_ denotes the total number of records in the dataset, row r and column c.


$$\begin{array}{l} Standard\;deviation\;\left(s_{d}\right)=\sqrt{\frac{{\left(P_{R}-M_{n}\right)}^{2}}{N_{R}}} \end{array}$$ (2)

The above equation estimates the standard deviation (*s*_*d*_) based on present records and mean values.


$$\begin{array}{l} Z_{score}=\frac{P_{R}-M_{n}}{s_{d}},\;s_{d}\neq 0\;and\;P_{R}-M_{n},s_{d}=0 \end{array}$$ (3)

The equation above is used to identify irrelevant values using *Z*-scores based on the mean and standard deviation values.


$$\begin{array}{l} Pre_{D}=\overset{c}{\underset{r}{\sum }}\frac{Z_{score}-Minimum\left(F_{v}\right)}{Maximum\left(F_{v}\right)-minimum\left(F_{v}\right)} \end{array}$$ (4)

The above equation is used to normalize *Pre*_*D*_ and the records in the dataset based on *Z*-score values. Let us assume *F*_*v*_ refers to the minimum and maximum feature values from the collective dataset.

The PD dataset records are preprocessed to prepare for feature analysis. First, check if the record contains all data values. Filling, removing, and ensuring data reliability are performed in a noisy dataset. All checks are complete, and each record works within defined margins. Checks for the existence of all registry values associated with property attributes. To facilitate PD diagnostics, it is necessary to preprocess the dataset by organizing and manipulating records to reduce its dimensionality.

This technology supports the development of Algorithm 1 for noise reduction while preserving the original data. In the process of categorization, the vast amount of data and incremental enhancements can result in a time-consuming task.

Algorithm 1 Z-score normalization-based dataset pre-processing for PD dataset. 
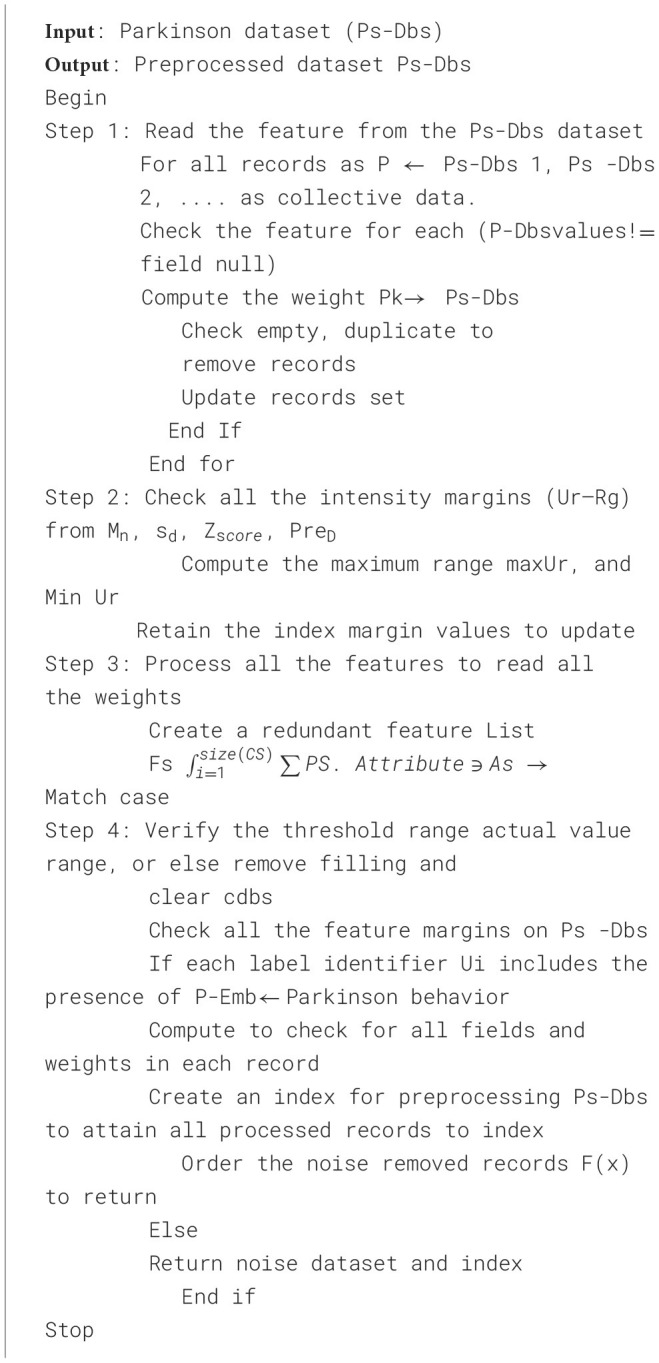

On a positive note, the remaining portion of the unprocessed dataset is now ready for PD diagnosis. It is important to acknowledge that unsupported data within the dataset, often exhibiting atypical properties, is characterized by fragility and noise.

**Figure 3:**
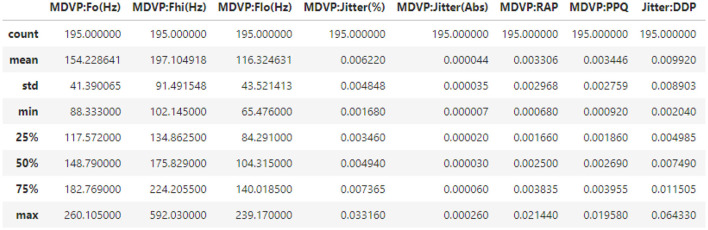
Screenshot for data pre-processing.

Figure 3 shows the simulation results for data pre-processing using the ZSN method. This technique eliminates missing and irrelevant values from the collected dataset. This section efficiently finds missing and irrelevant values in the dataset for PD prediction.

### 3.3 Disease affect scaling rate (DASR)

After data preprocessing, the DASR technique is used to identify the disease feature weight. Feature importance denotes a class for assigning weights to predictive model input features. This method identifies the PD weight and emphasizes which features are related to disease prediction. Therefore, the most crucial feature weight is calculated from the prediction of the preprocessed data set.


$$\begin{array}{l} P_{C}=F\left(x\right)\frac{Pre_{D}\left(A_{1}-A_{2}\right)}{B_{A}\sqrt{\frac{1}{Pre_{D}\left(A\right)}}} \end{array}$$ (5)

The equation above analyzes the two classes for each feature in the dataset *P*_*C*_. Assuming that *A* denotes attributes in the dataset and *B*_*A*_ refers to key features.


$$\begin{array}{l} B_{A}=\frac{{Pc.A}_{1}n_{1}^{2}+A_{2}n_{2}^{2}}{A_{1}+A_{2}-2} \end{array}$$ (6)

From Equation 6, it is possible to estimate significant features B^A^. Assuming that $n_{1}^{2}\;$and $n_{2}^{2}\;$denoting variance.


$$\begin{array}{l} W_{g}=\sum \frac{P_{C}\left(B_{A}\right)}{T_{A}} \end{array}$$ (7)

The above equation estimates the weight W_g_ of features in the preprocessed dataset. The dataset is assumed to have TA attributes.


$$\begin{array}{l} Finest\;feature\;\left(F^{2}\right)=W_{g}^{*}E_{rate}+\left(1-W_{g}\frac{A}{T_{A}}\right) \end{array}$$ (8)

An equation is used to evaluate the Finest Features *F*^2^ for feature selection. Assuming that E_rate_ denotes an error rate. This section provides a comprehensive explanation of Parkinson's disease's most prominent feature, weight.

**Figure 4:**
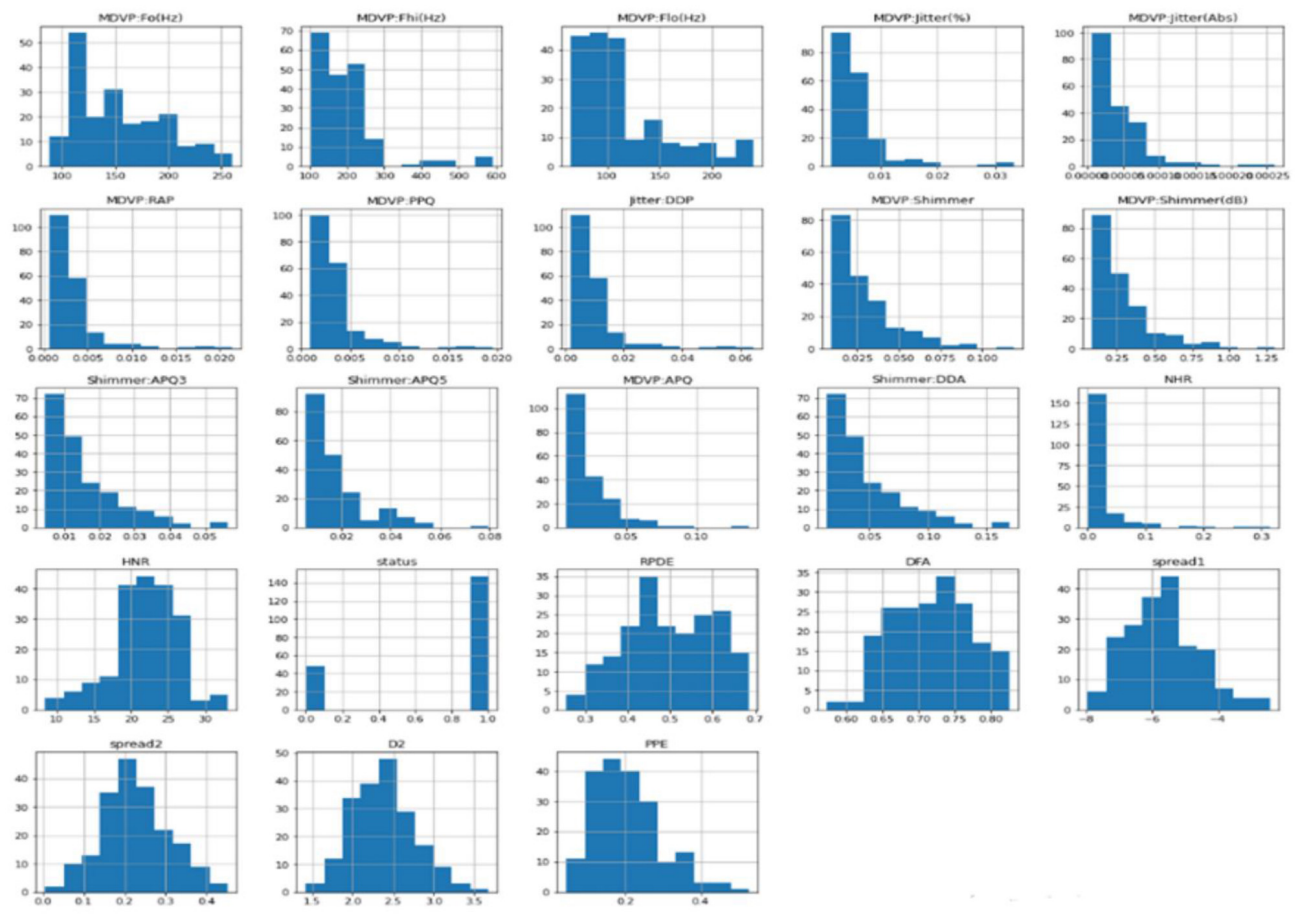
Screenshot for feature scaling rate.

Figure 4 shows the feature scaling rate used to identify the critical feature set for PD. This reveals the highest feature scaling rate and suggests that these features are essential for diagnosing Parkinson's disease.

### 3.4 Logistic decision exhaustive feature selection (LDEFS)

After feature weight analysis, we applied the LDEFS technique. It is proposed that the activity's preferred method be reduced to facilitate the analysis and processing of the input dataset. This is done to find the most appropriate attributes of disease prediction information. The proposed method quantifies uncertainty (information content) using entropy. Gain information measures the decrease in uncertainty compared to PD classification. This relationship is transformed into a probabilistic criterion in the logistic decision to choose the most impactful features. Using the logistic method, the main characteristics of the disease are selected.

Algorithm 2 describes how to obtain optimal PD prediction features based on the LDEFS technique. Initially, estimate the entropy values evaluated, then calculate the disease feature gain information, GInfor. Afterwards, the important features in the processed dataset will be evaluated. Next, we obtain optimal features based on logistical decisions. It selects the most appropriate values for the disease when the feature value exceeds the threshold value; otherwise, it ignores it. Assuming that P refers to probability, Ex refers to expected feature values, and the threshold value of 0.85. The threshold is 0.85 corresponds to features with a high logistic decision correlation. The features at this level and below will be weaker relative to the model's decision boundary.

Algorithm 2 Logistic decision exhaustive feature selection (LDEFS) for optimal feature extraction. 
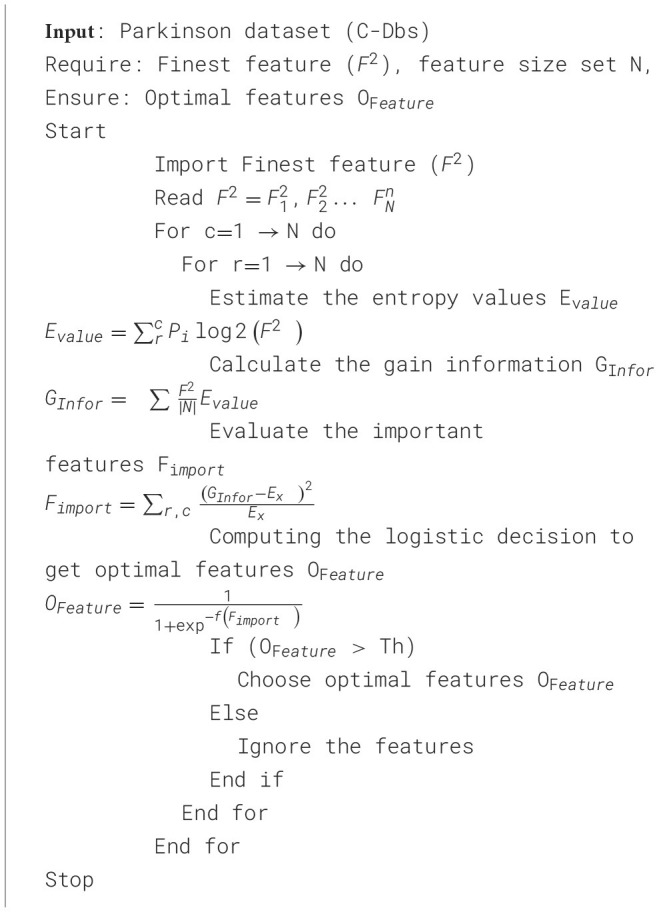

**Figure 5:**
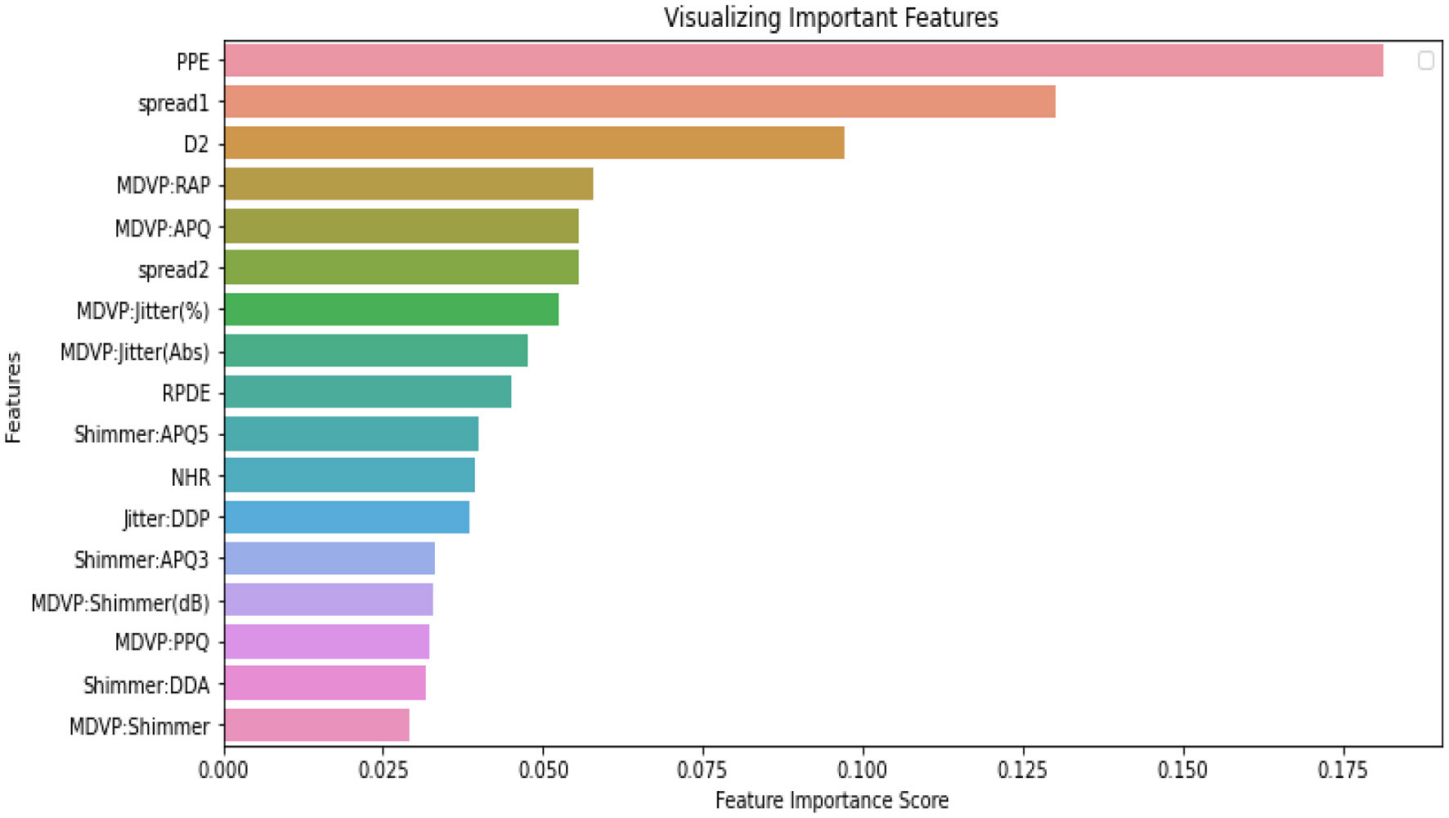
Identifying the important features.

Figure 5 presents the features with important scores for PD detection using the LDEFS technique. The most relevant features are Pitch Period Entropy (PPE), spread1, D2, and MVDP RAP.

### 3.5 Mamdani fuzzy neural network

After optimal feature selection, we explored the MFNN classification technique for PD prediction using a threshold-based feature selection class. The proposed mechanism categorizes PD based on If-Then rules. It has four layers of rules. In the first layer, the selected feature values are sent as input to the MFNN.

**Figure 6:**
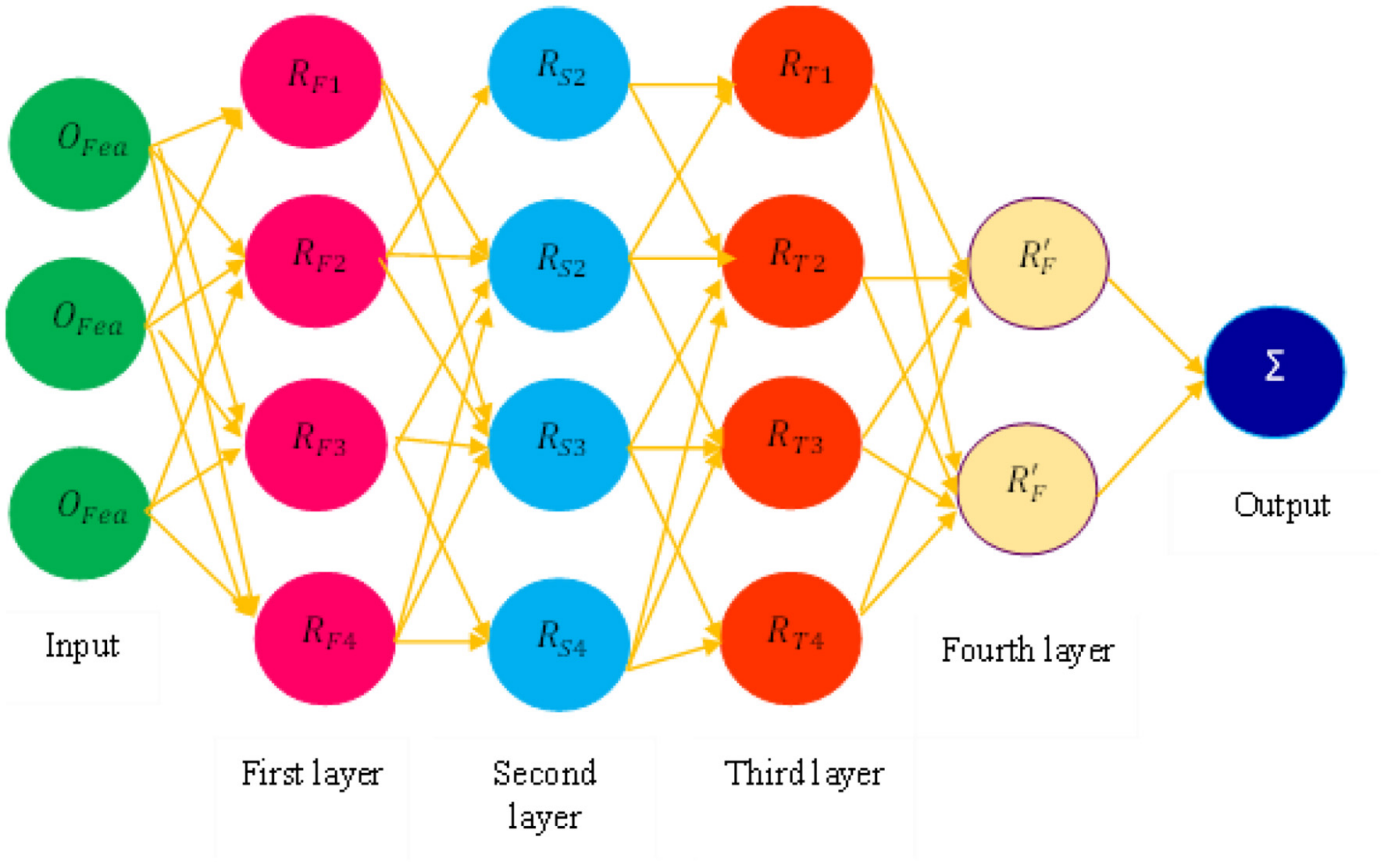
Process of Mamdani fuzzy neural network (MFNN).

Figure 6 defines the process of the MFNN for PD prediction. Each layer has rules for disease prediction, and these layers extract essential features to reduce feature dimensions. The weight parameter is fine-tuned using the membership function. Finally, the layers are included in the decision-making process.


$$\begin{array}{l} \omega =\tau \text{Δ}W_{g}+\alpha W_{g}\left(n_{w}+1\right) \end{array}$$ (9)

Equation 9 is used to update the feature weight ω, assuming τ, the learning rate range, is 0.4–0.8, and the selected learning rate tunes the weight values to achieve efficient disease prediction results. nw refers to a new weight and denotes a positive coefficient.

The MFNN algorithm's vital rule for disease prediction is as follows,

Rule 1:


$$\begin{array}{r} If\;O_{Feature1}\;is\;M_{i}\;and\;O_{Feature2}\;is\;L_{i}\\ then\;R_{i}p_{r}=p_{rO_{Featurei}}+q_{r+1}O_{Featurei+1}+z_{i} \end{array}$$ (10)

Rule 2:


$$\begin{array}{r} If\;O_{Feature1}\;is\;M_{i+1}\;and\;O_{Feature2}\;is\;L_{i+1}\\ then\;R_{i}p_{r+1}=p_{r}{O_{Featurei+1}+q_{r+1}O}_{Featurei+1}+z_{i+1} \end{array}$$ (11)

Where M_i_, L_i_, M_(i+1)_ and L_(i+1)_ is the fuzzy set; O_Feature1_ and O_Feature1_ optimized features; p_r_, q_r_, z_i_, p_(r+1)_, q_(r+1)_ and z_(i+1)_ predicted parameters.

The algorithm's steps 2 are in the MFNN classifier for Parkinson's disease.

Algorithm 3 explains the PD classification based on four-layer rules using the MFNN algorithm. Let us assume μ_*i*_ membership function and *O*_*Featurei*_ input to the node.

Algorithm 3 Mamdani fuzzy neural network (MFNN)-based Parkinson's disease classification rules. 
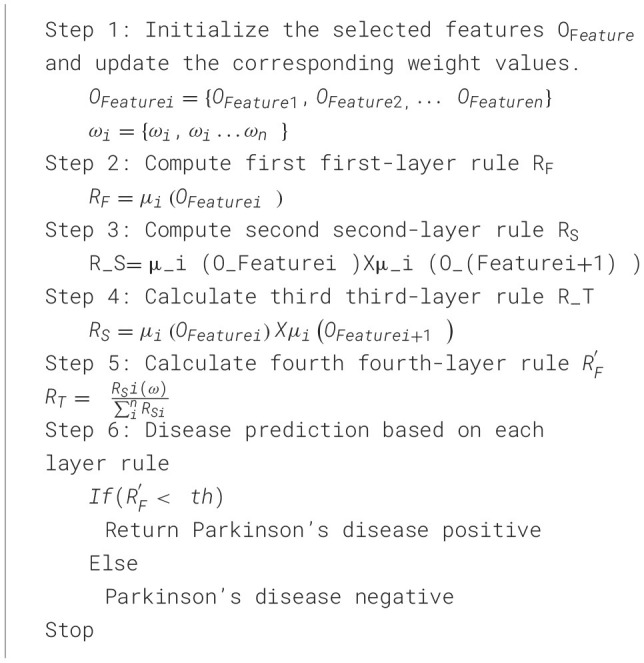

Here we calculate


$$\begin{array}{l} \mu_{i}=\exp\left(-\frac{|M_{i}-L_{i}{|}^{2}}{2{z_{i}}^{2}}\right) \end{array}$$ (12)

Moreover, the resultant features are identified using ideal margins from the relevance rate based on the finest feature limits. To match the PD rate intensity, select the relevant features.

The relationship between each feature and the optimal feature f_b_, derived from the pattern F_p_, is restrained, and the article weight f_b_ is examined. Atlas, the supreme attribute of heaviness designs, is organized and made the f_b_ done restatement in Algorithm 4. The algorithm uses a mutual information sorting method to determine features that best correlate with Parkinson's disease. The fuzzy membership functions are generated using the final feature subset (f_b_) in the MFNN, enabling efficient and interpretable risk classification of PDs. The remarkable one-way process maximizes classification performance by minimizing noise, redundancy, and overfitting.

Algorithm 4 Mutual information-driven finest feature extraction for MFNN input generation. 
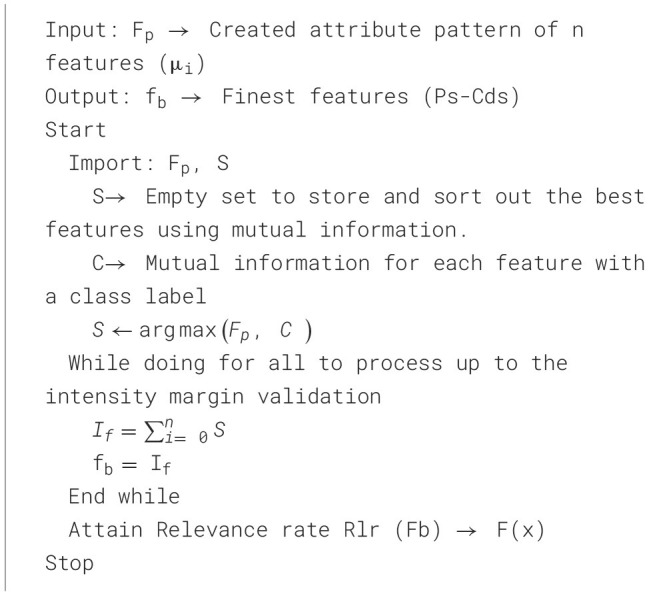

The Algorithm 5 process classifies the trained PD's classes based on the logical features of the evaluated training set. A neural classifier for premature treatment predicts recommendations based on disease prevalence and disease-affected rates. This algorithm identifies patients at risk for PD.

Algorithm 5 Risk-based Parkinson's Disease class assignment using MFNN prediction. 
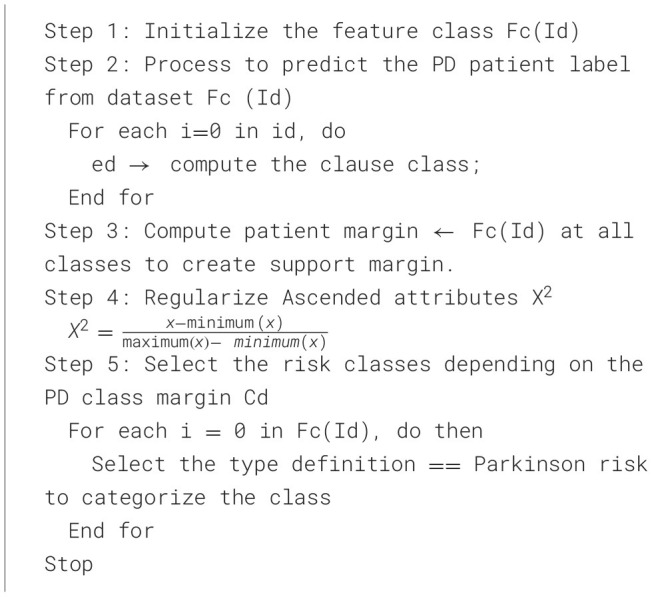

**Figure 7:**
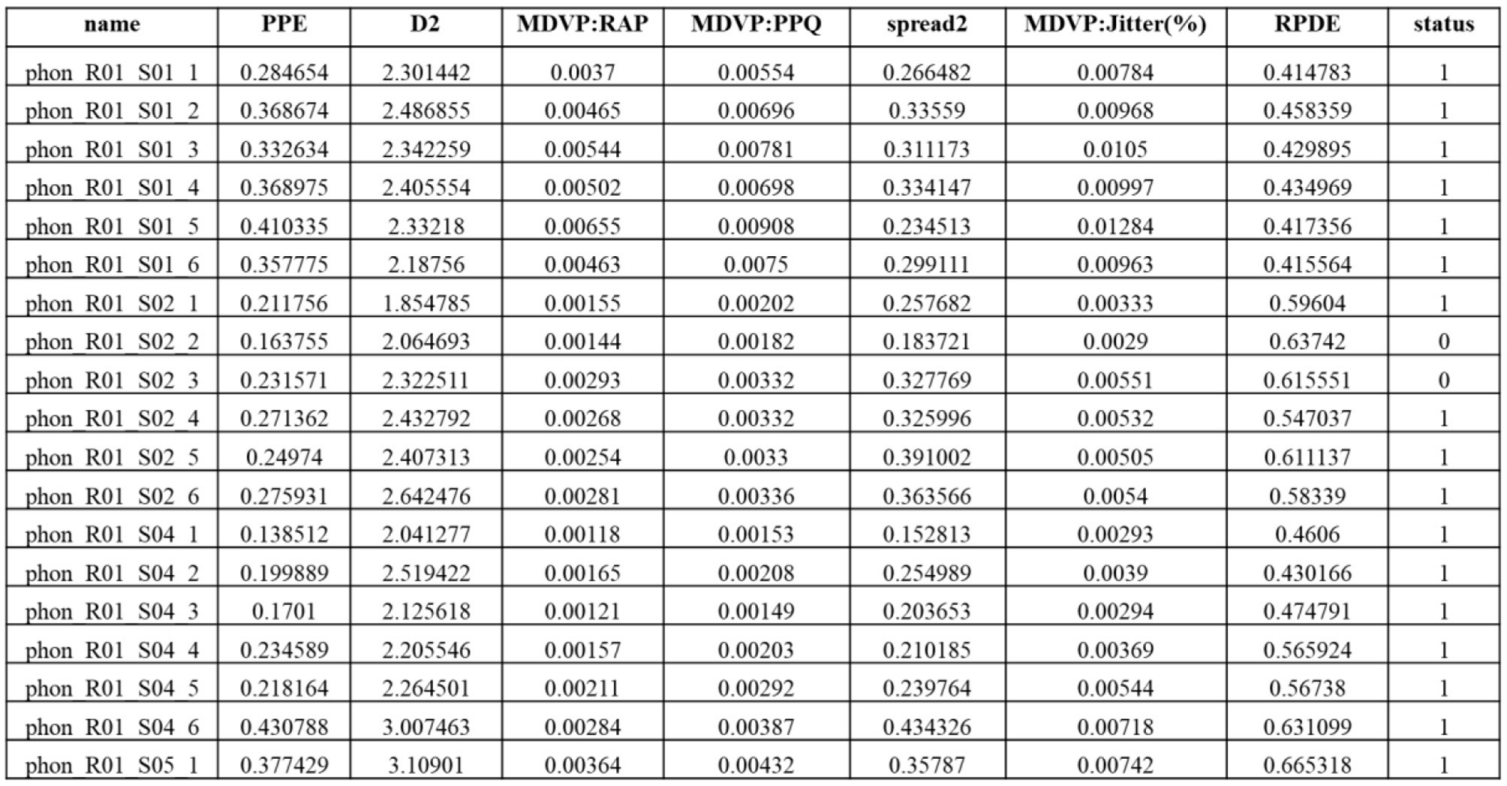
Simulation screenshot for Parkinson's disease classification.

Figure 7 shows the PD classification results using the MFNN algorithm.

## 4 Results and discussion

This section presents an analysis of the efficiency of the proposed implementation using a PD dataset obtained from an online repository. It contains simulation environment setup, performance evaluation, and comparison models for disease prediction.

### 4.1 Simulation environment setup

The simulation experiment was performed using Python 3.11.1 on 64-bit Windows 10, with an Intel Core i5 2.20 GHz processor and 16 GB of RAM. This study used a confusion matrix to examine the resulting parameters and version. A method is proposed for selecting features based on maximum support weights. Based on the confusion matrix, high validation levels are applied to the training features. The MFNN process performance is analyzed and measured using various parameters. These samples are taken from human lymphoma marrow models. These features are tested by testing medical margin values.

Table 1 defines the detailed simulation parameters for PD prediction. This research uses the Parkinson's disease dataset.

**Table 1:** The model for simulation parameters.

**Limitation**	**Values**
Tool	Anaconda
Language	Python
Name of the dataset	Parkinson's disease dataset
No. of records	4,000
Training	60%
Testing	20%
Validation	20%

### 4.2 PD dataset description

Parkinson's disease datasets were collected at the Kaggle repository; the dataset employed in this study consists of attributes collected from 500 people with PD symptoms. It includes 24 features and random samples of 500 records from the gathered dataset presented in Table 2.

**Table 2:** Dataset details and records.

**Dataset name**	**Features**	**No. of records**	**Normal**	**Abnormal**
PD-Ds dataset	24	5,113	2,456	2,657
PD-Ds-UPDRS	22	4,108	1,437	2,671
PPMI data	18	5,173	3,281	1,892
PD-fox insight	21	6,906	5,234	1,672

### 4.3 Performance evaluation metrics

The performance metric evaluated for the proposed method is its ability to distinguish healthy subjects from PD patients. To reach this estimate, we need to measure four key factors.


$$\begin{array}{l} Precision=\frac{\sum True_{positive}}{\sum True_{positive}+False_{positive}}*100 \end{array}$$ (13)

$$\begin{array}{l} Recall=\frac{\sum True_{positive}}{\sum True_{positive}+False_{negative}}*100 \end{array}$$ (14)

$$\begin{array}{l} F1-score=\sum 2X\frac{Pre*Rec}{Pre+Rec}*100 \end{array}$$ (15)

$$\begin{array}{l} Accuracy=\\ \frac{\sum True_{positive}+True_{negative}}{\sum True_{positive}+False_{negative}+False_{positive}+True_{negative}}*100 \end{array}$$ (16)

**Figure 8:**
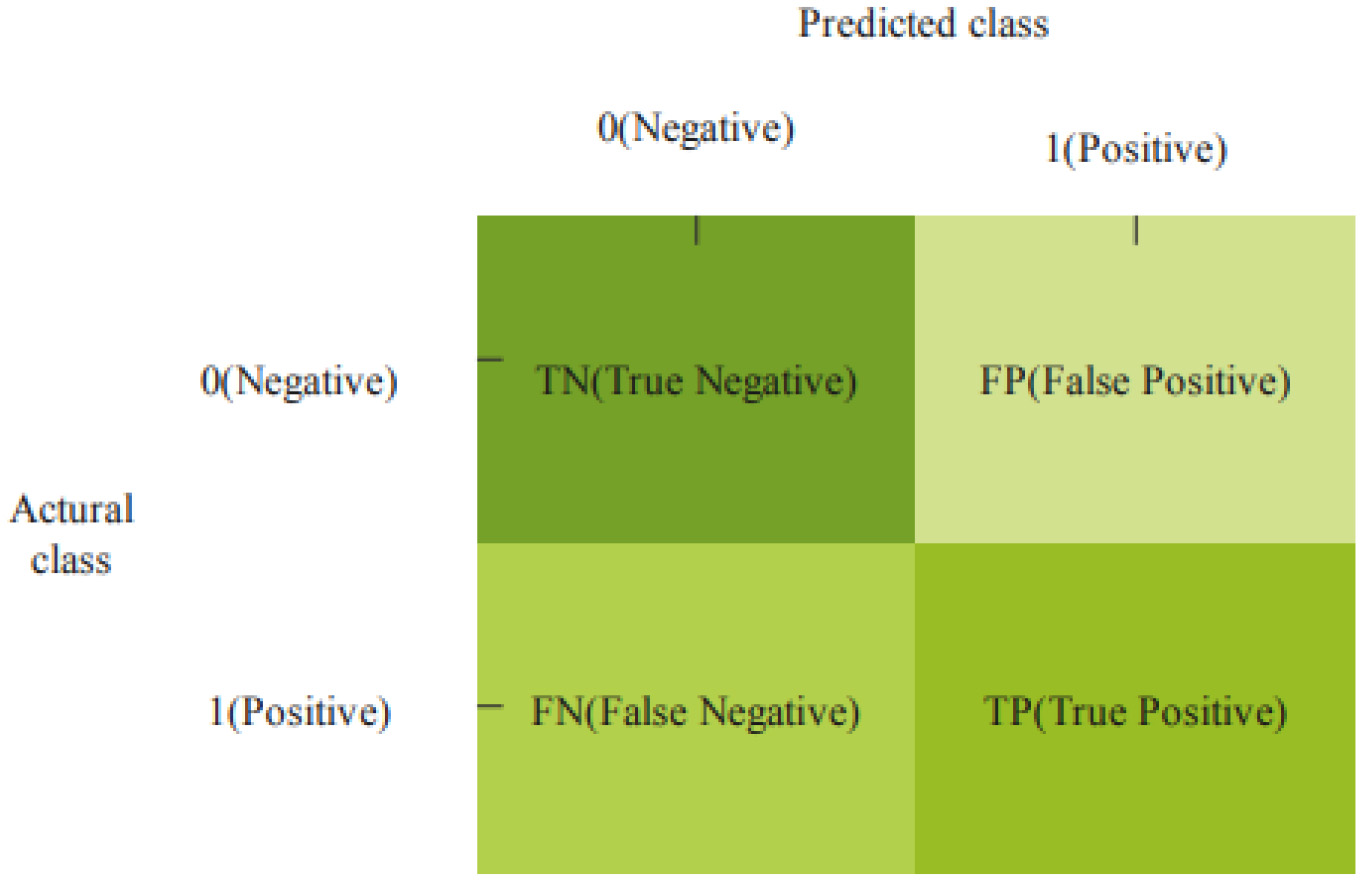
Confusion matrix.

Figure 8 presents the confusion matrix to predict PD, TN, TP, FP, and FN.

### 4.4 Comparison model for PD–Ds dataset

SVM and ANFIS are currently the most popular algorithms. The proposed MFNN, as a novel approach, will enhance existing algorithms, improving their accuracy and efficiency. This is done by incorporating various features and producing an optimized result.

**Figure 9:**
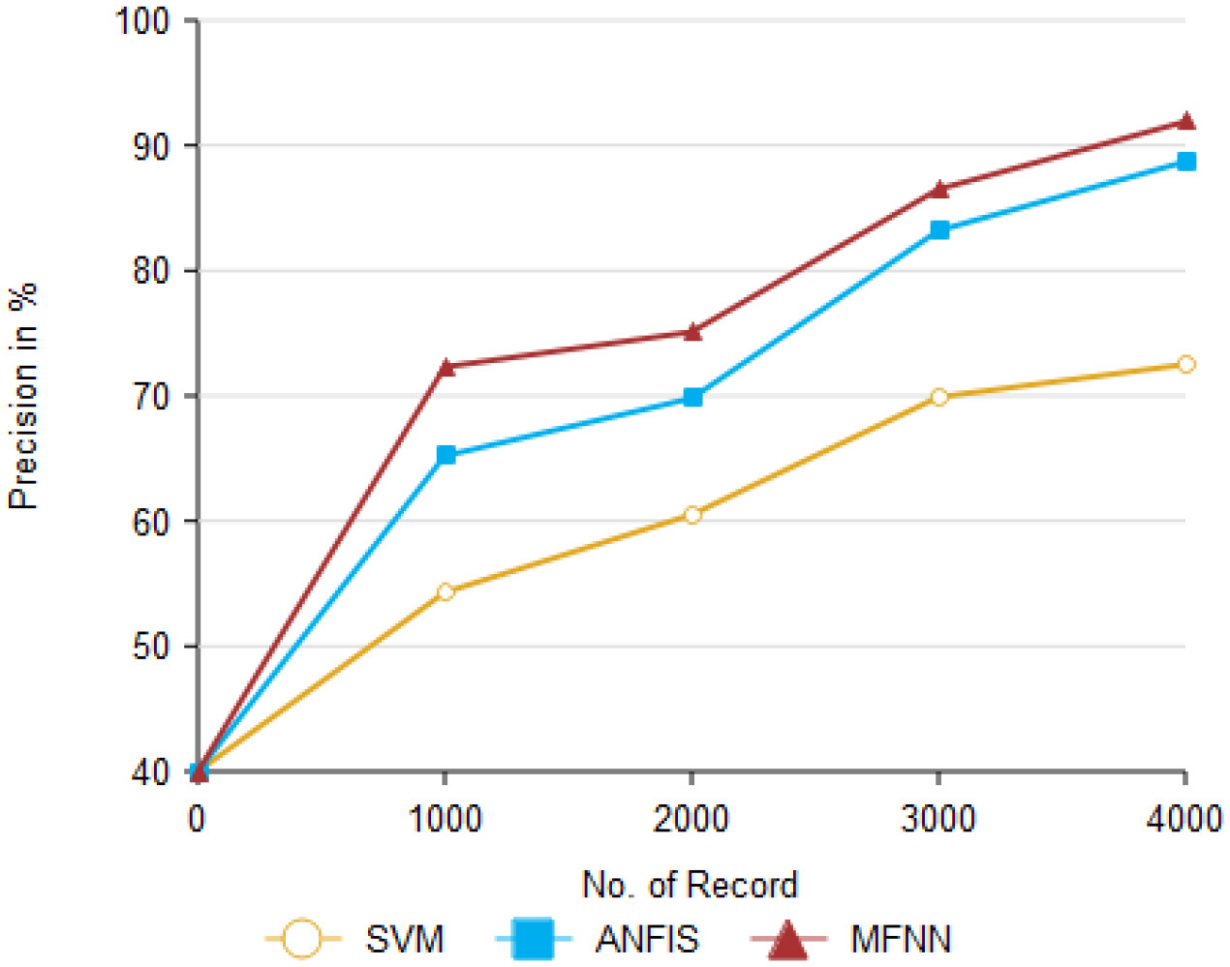
Precision performance.

Figure 9 and Table 3 present the precision performance of the proposed MFNN method compared to previous ML classifiers. Precision is a measure of the accuracy of positive predictions in the context of predicting Parkinson's disease. It indicates the proportion of correct positive predictions made by the model compared to the total number of positive predictions. The proposed MFNN method achieved 91.9% higher precision on 4,000 records than other methods. In comparison, the existing methods, SVM and ANFIS, had 72.5% and 88.7% precision, respectively.

**Table 3:** Analysis of precision performance.

**Comparison methods/No. of records**	**SVM**	**ANFIS**	**MFNN**
1,000	54.3	65.2	72.3
2,000	60.5	69.8	75.1
3,000	69.9	83.2	86.5
4,000	72.5	88.7	95.9

**Figure 10:**
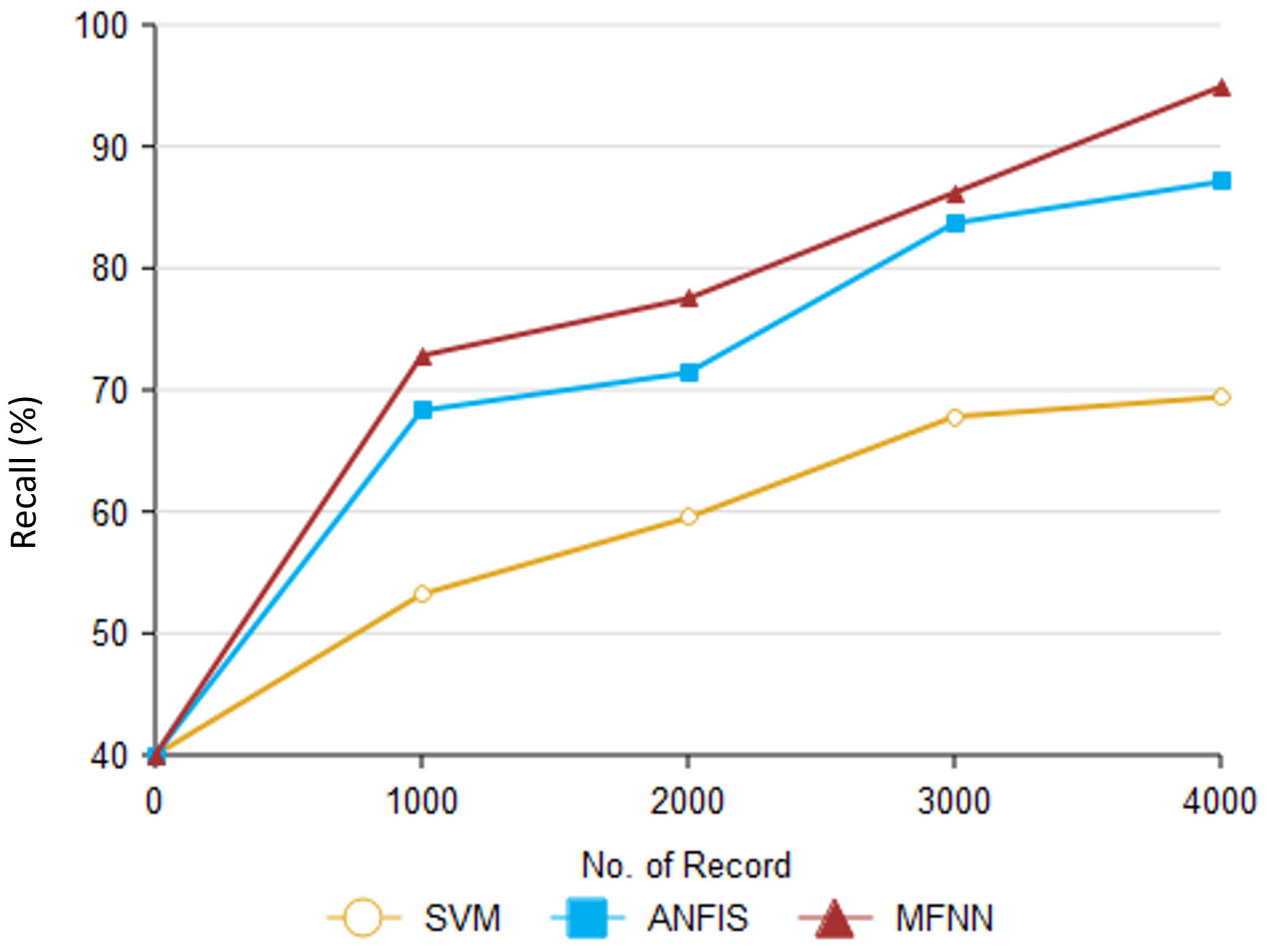
Result of recall performance.

Figure 10 and Table 4 show that recall outperforms different ML methods for PD prediction. The proposed MFNN algorithm for predicting PD involves identifying true positives and false negatives. True positives refer to the actual cases of PD that the proposed method accurately determined. At the same time, false negatives are cases of PD that were incorrectly identified as non-Parkinson's. The proposed MFNN algorithm has 95.3% recall performance, whereas the existing system has 87.1%, and SVM has 69.4% recall performance for 4,000 records.

**Table 4:** Result of recall performance.

**Comparison methods/No. of records**	**SVM**	**ANFIS**	**MFNN**
1,000	53.2	68.3	72.8
2,000	59.5	71.4	77.5
3,000	67.8	83.7	86.2
4,000	69.4	87.1	94.9

**Figure 11:**
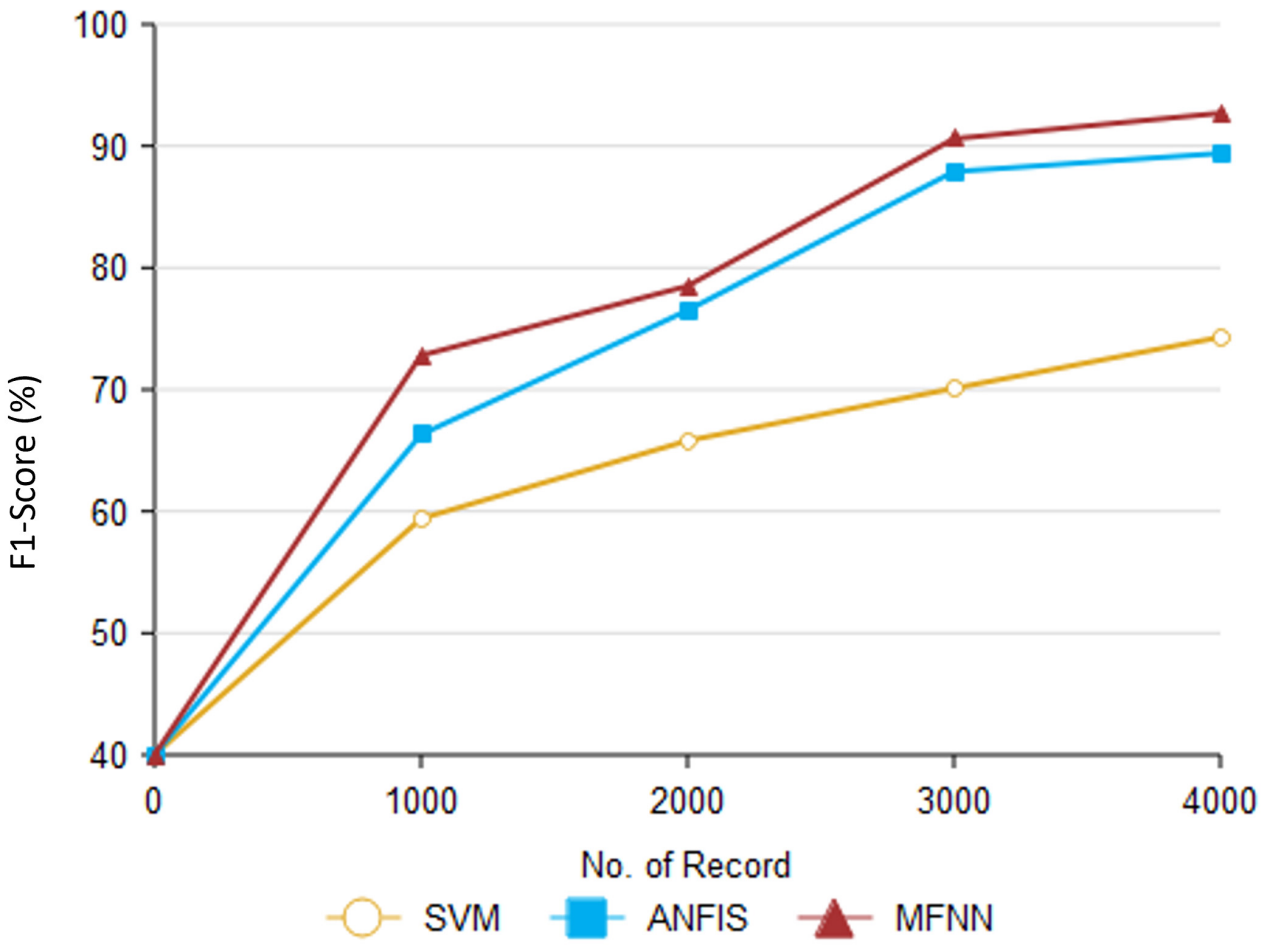
Result of F1-score performance.

Furthermore, Table 5 and Figure 11 present F1-score performance with various classification methods. In predicting PD using the MFNN method, the F1 score is used to evaluate the proposed performance in terms of both precision and recall. It provides a balanced evaluation of the proposed performance, indicating how well it makes accurate positive predictions while capturing a high proportion of actual positive instances. The proposed MFNN algorithm achieved the highest F1-score for predicting PD, outperforming the other methods. In comparison, SVM achieved 74.3% and ANFIS reached 89.4% on the same dataset consisting of 4,000 records.

**Table 5:** Analysis of F1-score.

**Comparison methods/No. of records**	**SVM**	**ANFIS**	**MFNN**
1,000	59.4	66.3	72.8
2,000	65.8	76.5	78.5
3,000	70.1	87.9	90.6
4,000	74.3	89.4	95.3

The MFNN method's ability to correctly identify Parkinson's cases and distinguish them from non-Parkinson's cases is evaluated through classification to assess its predictive performance for Parkinson's disease.

**Figure 12:**
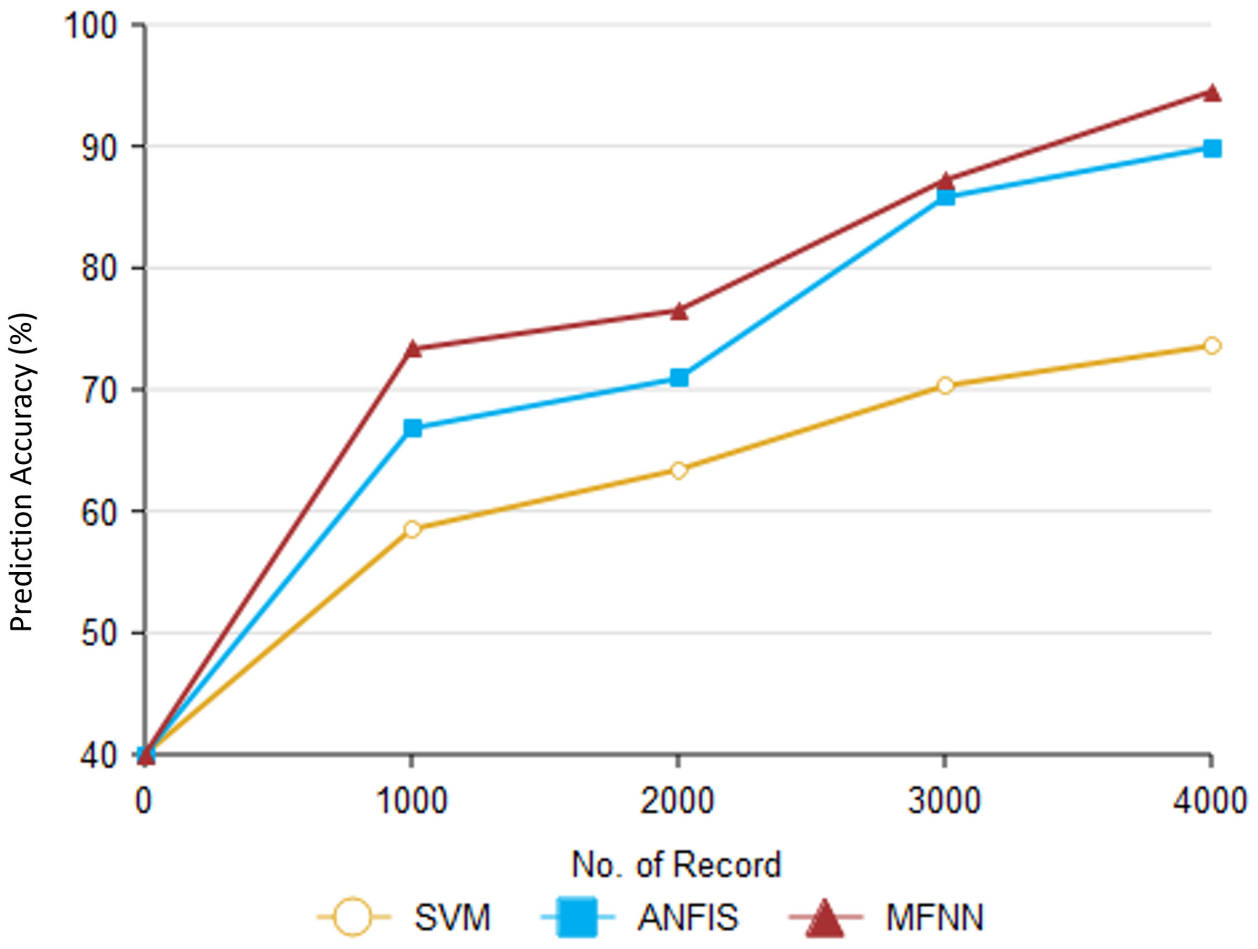
Classification performance by risk levels.

Figure 12 and Table 6 describe the overall MFNN method to predict PD in various records. It can handle 4,000 records and achieve PD classification accuracy of 95.8% with the proposed MFNN algorithm. The classification results improved as the dataset size increased. Similarly, the traditional SVM method classification result is 73.6%, and the ANFIS method classification result is 89.9%.

**Table 6:** Result of classification performance for analysis.

**Comparison methods/No. of records**	**SVM**	**ANFIS**	**MFNN**
1,000	58.5	66.8	73.3
2,000	63.4	70.9	76.5
3,000	70.3	85.8	87.2
4,000	73.6	89.9	95.8

Based on various classification methods, Table 7 presents false classifications that have been produced. According to the proposed MFNN algorithm, 5.4% of predictions for PD are false positives. The proposed method has a lower false-rate performance than the existing method. This indicates that the MFNN algorithm is more reliable in predicting PD. Furthermore, the MFNN algorithm is more cost-effective and efficient than the existing method.

**Table 7:** Exploration of false classification performance.

**Comparison methods/No. of records**	**SVM**	**ANFIS**	**MFNN**
1,000	28.9	13.8	13.1
2,000	25.6	12.4	10.8
3,000	22.6	11.5	7.6
4,000	16.2	10.6	5.4

**Figure 13:**
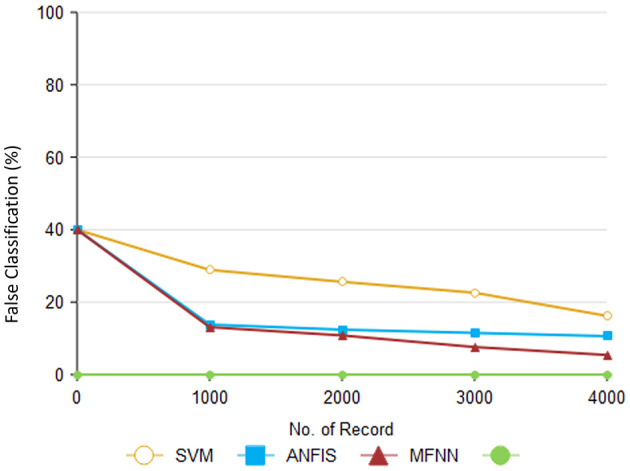
Analysis of false classification ratio.

Figure 13 illustrates how the proposed method compares to previous methods. According to Table 8, different methods are accompanied by different companions. As part of the proposed approach, unwanted records are eliminated from the dataset through preprocessing. The SVM method's performance was 16.2%, and the ANFIS method's performance was 10.6%.

**Table 8:** Comparison of different methods.

**Dataset**	**Methods**	**MFNN**			**ANFIS**			**SVM**		
	**Testing validation in %**	**Precision in %**	**Recall in %**	**Accuracy in %**	**Precision in %**	**Recall in %**	**Accuracy in %**	**Precision in %**	**Recall in %**	**Accuracy in %**
PD-Ds dataset	95.5	95.8	92.2	95.8	83.2	84.3	84.2	81.2	80.3	80.1
PD-Ds-UPDRS	94.3	95.3	94.6	93.8	81.4	82.2	82.5	79.1	80.2	81.2
PPMI data	96.1	96.3	95.3	95.2	82.5	84.3	85.2	80.4	78.1	79.2
PD-fox insight	95.2	94.2	95.4	93.8	83.6	84.1	85.2	80.8	79.7	80.9

The DASR is used to analyze the related features of Parkinson's risk level. Based on the marginal rate, the features are divided into class labels. Furthermore, we proposed a classifier and feature selection method that produces a false classification performance of 5.4%, which is lower than the false classification performance produced by other methods.

**Figure 14:**
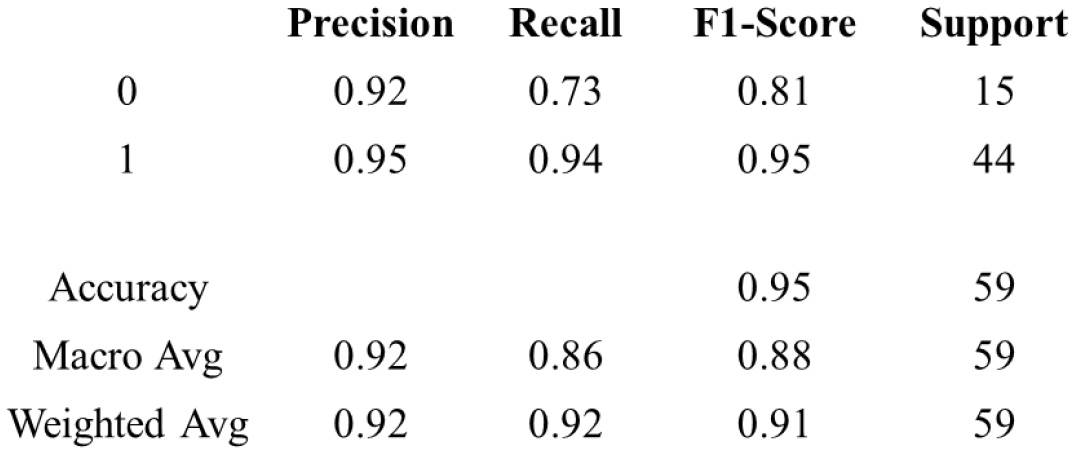
Result of classification performance.

Figure 14 discusses PD prediction and classification, along with the pathology. The MFNN model, as proposed, demonstrated a precision rate of 0.95, a recall rate of 0.94, an F1-score of 0.95, and an accuracy rate of 0.95.

**Figure 15:**
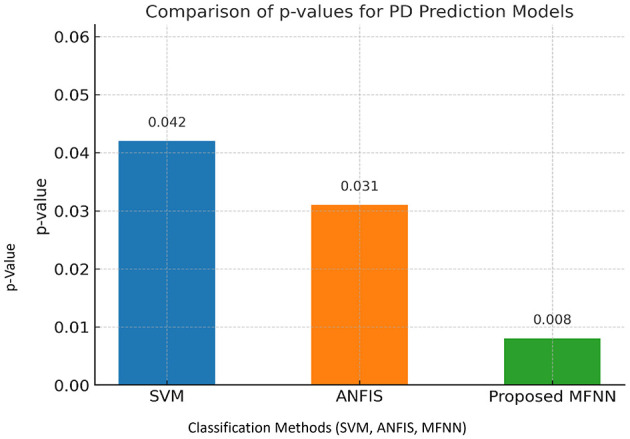
Parkinson's disease prediction accuracy.

An illustration of the accuracy of risk prediction is shown in Figure 15. The proposed method efficiently generated patient risk levels based on the collective dataset. Risk classes are classified based on a risk threshold to prove the results. This achieves very high prediction accuracy.

**Figure 16:**
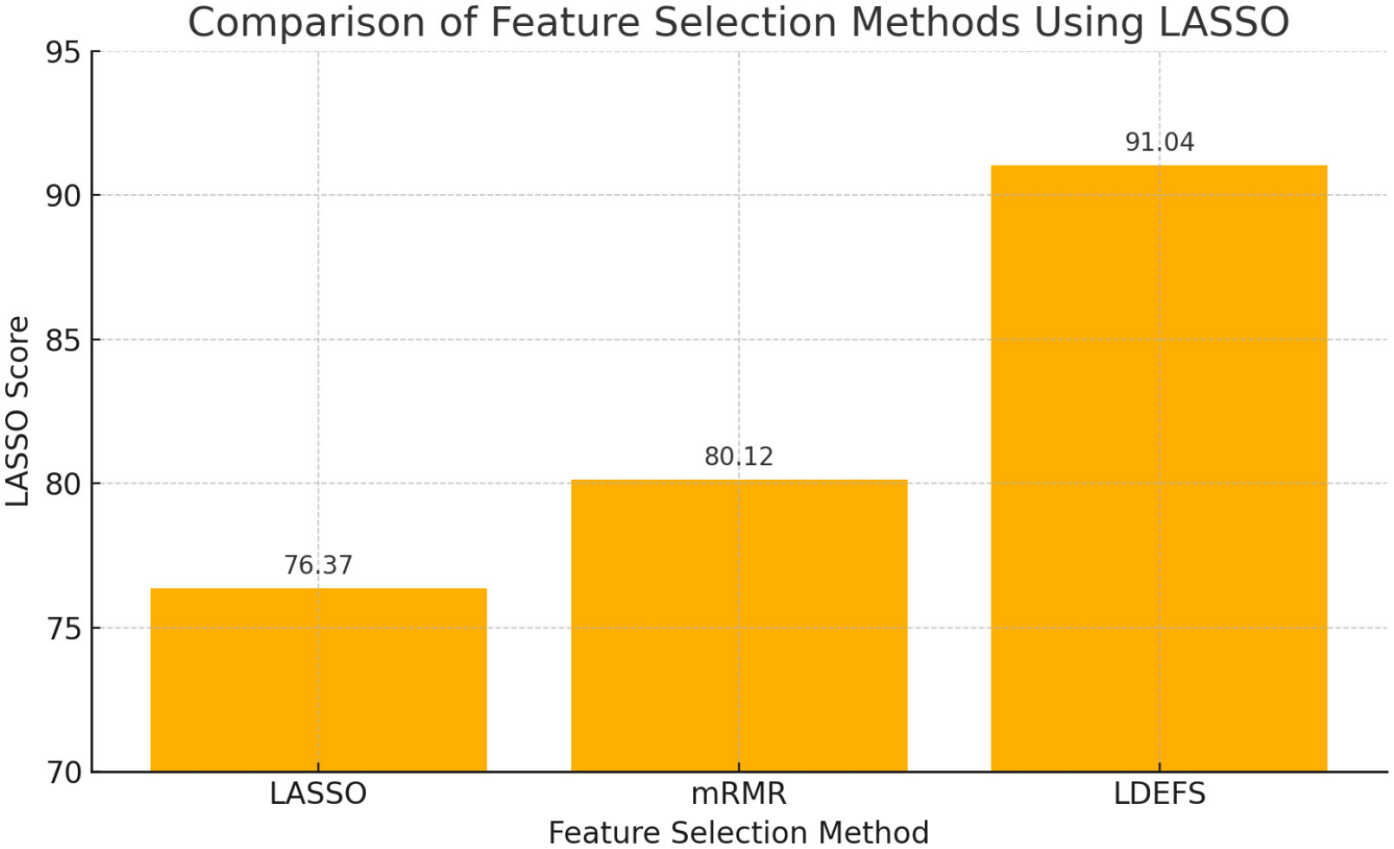
Analysis of feature selection performance.

Figure 16 demonstrates the feature selection performance for PD prediction with different methods. The proposed method attains 91.04% of feature selection performance, while the traditional methods, Least Absolute Shrinkage and Selection Operator (LASSO) and Minimum Redundancy Maximum Relevance (mRMR), obtained 76.37% and 80.12%, respectively.

**Figure 17:**
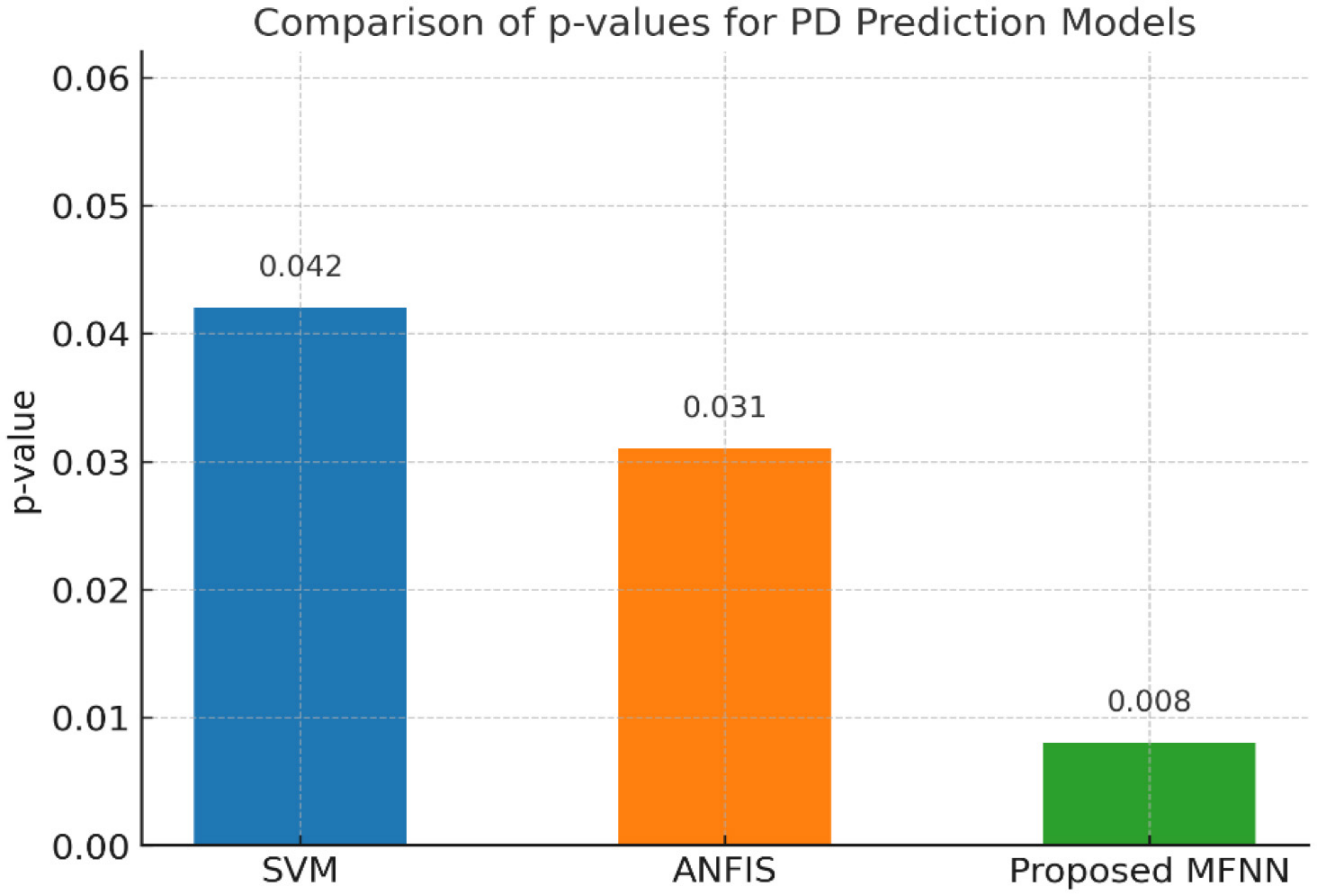
Performance of *p*-values.

Figure 17 demonstrates the performance of the *p*-value comparison result. The lowest *p*-value (0.008) was in the proposed MFNN model. Here, the statistical significance of the MFNN results is high, indicating that it is more effective and its predictions are less prone to coincidence. The SVM model is the least significant model with the highest *p*-value (0.042). ANFIS performs quite well, with a *p*-value that is higher than the SVM but lower than the MFNN model.

## 5 Conclusion

This study presented the ML-based LDEFS and MFNN approaches for predicting Parkinson's disease. The ZSN technique reduces noise and eliminates irrelevant records. Then, we applied the DASR technique to estimate the disease-feature weight. Using the disease-feature weight, we identified the most significant features of PD through the LDEFS technique. Our proposed MFNN algorithm with a softmax activation function predicted PD using the selected features. Experimental results demonstrated that our proposed system's accuracy outcome is 95.8% more effective than other systems. The precision rate is 95.3%, and the recall rate is 94.8%, higher than those of the other disease prediction systems.

## Data Availability

The original contributions presented in the study are included in the article/supplementary material, further inquiries can be directed to the corresponding author.
